# Dynamics of somatostatin 4 receptor expression during chronic-stress loading and its potential as a chronic-stress marker

**DOI:** 10.1038/s41598-024-58621-7

**Published:** 2024-05-02

**Authors:** Yuki Abe, Takehiko Murase, Masahide Mitsuma, Yoriko Shinba, Hiromi Yamashita, Kazuya Ikematsu

**Affiliations:** 1 Division of Forensic Pathology and Science, Department of Medical and Dental Sciences, Graduate School of Biomedical Sciences, School of Medicine, Nagasaki University, 1-12-4, Sakamoto, Nagasaki, 852-8523 Japan; 2 Division of Forensic Dental Science, Department of Medical and Dental Sciences, Graduate School of Biomedical Sciences, School of Medicine, Nagasaki University, 1-12-4 Sakamoto, Nagasaki, 852-8523 Japan; 3 Department of Forensic Medicine, Faculty of Medicine, Kagawa University, 1750-1, Miki, Kita, Kagawa, 761-0793 Japan

**Keywords:** Chronic stress, Restraint stress, Somatostatin receptor subtype-4, Biomarkers, Thymus gland, Molecular biology, Biomarkers

## Abstract

Chronic stress has been implicated in mental illnesses and depressive behaviors. Somatostatin 4 receptor (SSTR4) has been shown to mediate anxiolytic and depression-like effects. Here, we aimed to explore the potential of SSTR4 as a diagnostic marker for chronic stress in mice. The mice were divided into single stress, chronic restraint stress, and control groups, and *Sstr4* mRNA expression in the pituitary, lungs, and thymus, its protein expression in the thymus, were analyzed. Compared to controls, *Sstr4* mRNA expression decreased significantly in the pituitary gland of the chronic and single-stress groups (P = 0.0181 and 0.0022, respectively) and lungs of the single-stress group (P = 0.0124), whereas it significantly increased in the thymus of the chronic-stress group (P = 0.0313). Thymic SSTR4 expression did not decrease significantly in stress groups compared to that in the control group (P = 0.0963). These results suggest that SSTR4 expression fluctuates in response to stress. Furthermore, *Sstr4* mRNA expression dynamics in each organ differed based on single or chronic restraint stress-loading periods. In conclusion, this study suggests that investigating SSTR4 expression in each organ could allow for its use as a stress marker to estimate the stress-loading period and aid in diagnosing chronic stress.

## Introduction

Stress is a general psychophysiological response of the body to various threats (termed stressors). Chronic stress is a prolonged condition that can affect the organs and health of the living body^1^ and has recently been linked to arteriosclerosis, a potential cause of atherosclerotic cardiovascular and cerebrovascular diseases^2^. Chronic or multiple stress loads have also been shown to be associated with tumor occurrence and development^3^ as well as mental illness^4–7^.

Humans are often exposed to prolonged stress during intimate partner violence (IPV) and elder abuse, which have recently become major social issues. Yon et al*.*^8^ reported that one in six older adults worldwide experience abuse, including financial abuse. Moreover, the World Health Organization reported in 2013 that approximately 30% of ever-partners women would experience IPV during their lifetime^9^. Elder abuse and IPV are critical and potentially fatal issues^10,11^. For IPV, in particular, approximately one in seven homicides globally is committed by intimate partners^11^. Therefore, these problems are frequently encountered in forensic practice and require accurate diagnosis by forensic scientists.

As elder abuse and IPV victims have a strong and prolonged stress load, proof of chronic stress will be useful in such diagnoses. However, it is currently difficult to diagnose chronic stress in adults using forensic medicine. Thymus atrophy may be used to diagnose abuse and chronic stress in children^12^. However, it is not a definite verdict, particularly in the older population, as thymus atrophy is also observed with aging^13^. Therefore, thymus atrophy is not useful in diagnosing chronic stress in adults who may be victims of elderly abuse and IPV, and it is difficult to diagnose chronic stress in adults^12^.

Therefore, we focused on the Somatostatin 4 receptor (SSTR4). SSTR4 is a G-protein-coupled receptor that belongs to the SSTR family, which consists of five members (SSTR1-5). These proteins interact with somatostatin and exert their biological effects^14–16^. SSTR4 has been shown to mediate anxiolytic and depression-like effects. The potential of SSTR4 as a therapeutic target for anxiety and depressive disorders has also been reported^17–21^. In addition, chronic stress or multiple stress loads have recently been associated with mental illnesses, such as anxiety disorders, mood disorders, and depressive behavior, through various mechanisms^4–7^. Thus, it is possible that SSTR4 expression levels fluctuate during mental illness (such as anxiety and mood disorders) and depressive behavior caused by chronic stress, including IPV and elder abuse.

Kawashima et al*.*^22^ revealed that treating rat pituitary adenoma cells with high corticosterone concentrations for 48 h significantly increases *Sstr4* mRNA expression levels. Although glucocorticoids, including corticosterone, are secreted in rodents during stress loading^23^, they are not ideal markers of chronic stress because they respond to a single-stress event. However, considering that high glucocorticoid/corticosterone levels may persist during chronic-stress loading, *Sstr4* mRNA expression may be altered. Thus, *Sstr4* expression may change through multiple mechanisms during chronic-stress loading, making it a potential marker for diagnosing chronic stress.

Studies on SSTR4 and stress or depression-like behavior have only reported *Sstr4* mRNA expression in the brain, stress responsiveness and depression-like behavior in *Sstr4* -deficient mice, and the potential of *Sstr4* as a therapeutic target for anxiety and depressive disorders^17,21,24,25^.

Moreover, the kinetics of SSTR4 expression in the thymus and lungs during chronic stress are unknown. Thus, in the present study, we hypothesized that SSTR4 expression is affected by chronic stress. We examined the dynamics of *Sstr4* expression in the pituitary gland, which is assumed to be prone to *Sstr4* fluctuation under chronic stress; in the thymus, where changes (atrophy) caused by stress loading are observed; and in the lungs, where *Sstr4* expression has been confirmed in the Mouse Genome Informatics (MGI) database. In addition, we also examined SSTR4 expression in the thymus.

## Results

### Corticosterone concentration after restraint stress treatment

**Figure 1:**
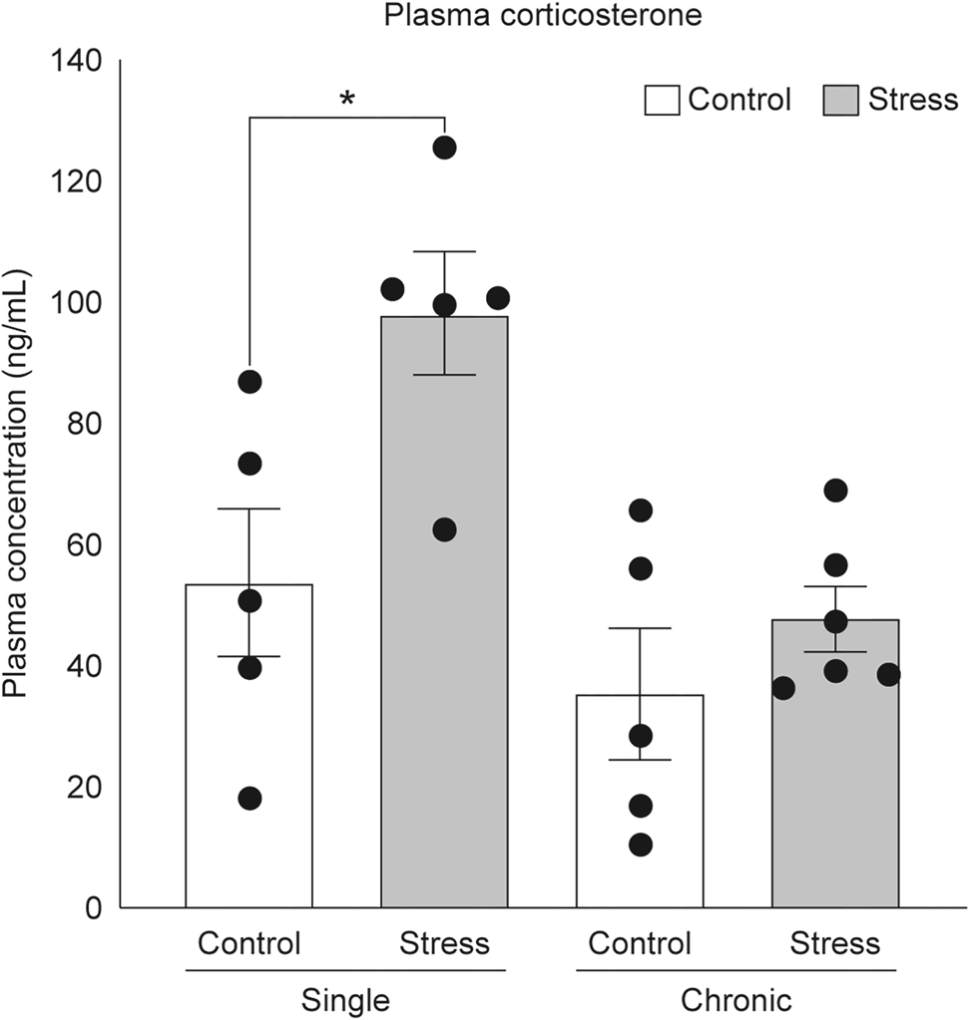
Plasma corticosterone levels after a single-stress event (Single) and chronic stress lasting one week (Chronic). The graph shows the plasma corticosterone concentration in each group. Data are shown as mean ± standard error, with n = 5–6 for each group. Individual data points are also shown in the graph. Significant differences between the single-stress group, chronic-stress group, and their respective control groups were investigated using Welch's t-test. *P < 0.05. Significant differences between the single-stress and chronic-stress groups were not investigated.

Plasma corticosterone concentration was significantly higher in the single-stress group than in the control group (P = 0.0241). No significant differences in corticosterone concentration were observed between the chronic-stress and control groups (Fig. 1).

### Changes in body and relative thymus weight

The chronic-stress group showed a significant decrease in the body weight of mice after stress treatment (P = 0.001, Table 1). In addition, the thymus weight and relative thymus weight were significantly lower in the chronic-stress group than in the chronic control group (P < 0.0001 and P < 0.0001, respectively, Table 1). There were no significant differences in body weight changes one day before euthanasia (after stress) or relative thymus weight between the single-stress group and its corresponding control group (Table 1).

**Table 1:** Mean and standard deviation of body weight change from before stress to the day before euthanasia (body weight after stress -body weight before stress), thymus weight, and relative thymus weight.

	Single control (n = 6)		Single stress (n = 7)			Chronic control (n = 6)		Chronic stress (n = 6)		
	Mean	SD	Mean	SD	P-value	Mean	SD	Mean	SD	P-value
Change in body weight	0.4633	0.4628	0.6943	0.3549	0.3440	0.8050	0.3174	− 3.6017	1.6457	0.001
Thymus weight (g)	0.0503	0.0079	0.0471	0.0089	0.5082	0.0524	0.0061	0.0159	0.0045	< 0.0001
Relative thymus weight (g/g body weight)	0.0020	0.0003	0.0018	0.0003	0.228	0.0020	0.0002	0.0007	0.0002	< 0.0001

For each group, n = 6–7. Significant differences between the single-stress group and its control group and between the chronic-stress group and its control group were investigated using Welch's t-test.

Additionally, no significant differences in body weight before stress were evident between the stress groups and their respective control groups (P = 0.3769 and P = 0.5824, respectively).

### Sstr4 mRNA expression

**Figure 2:**
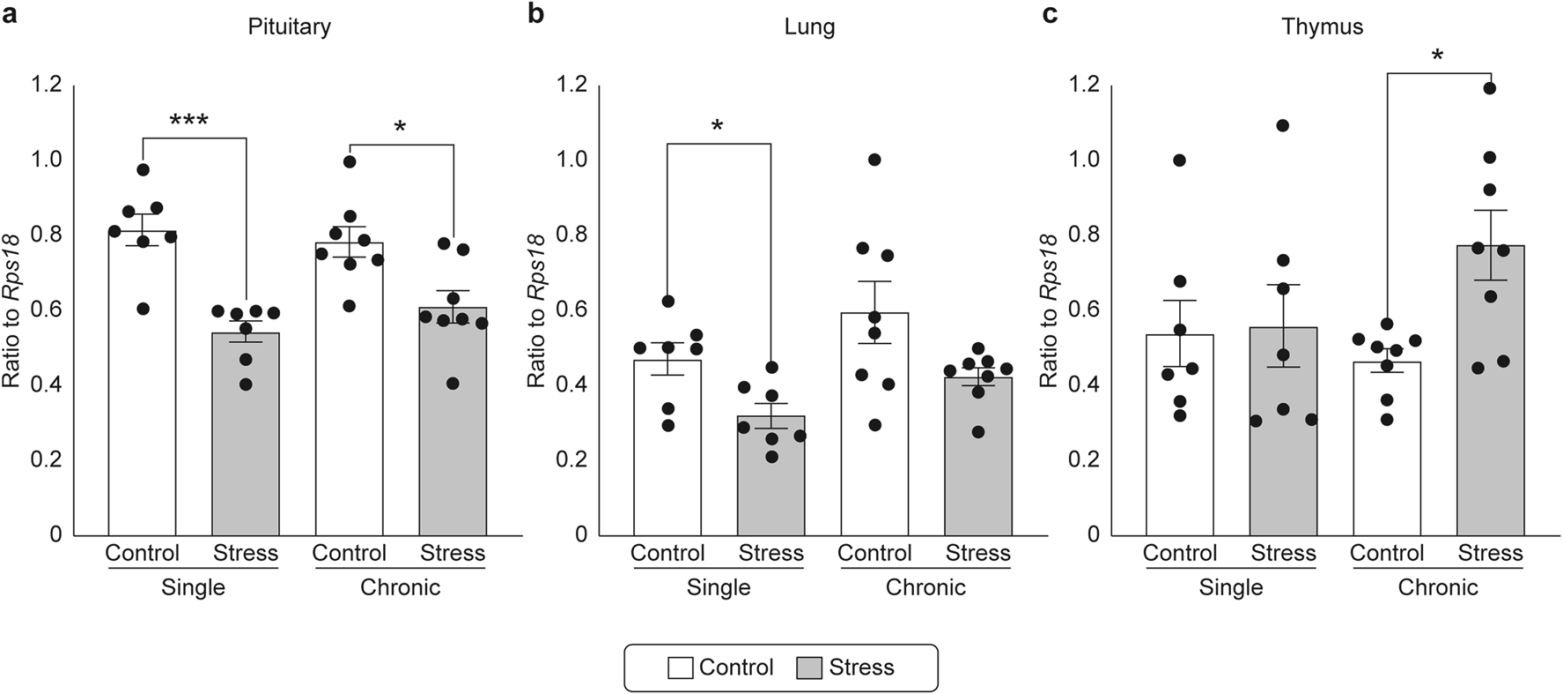
Changes in the dynamics of *Sstr4* mRNA expression in each organ for control, single-stress restraint, and chronic restraint stress load mice. (**a**–**c**) changes in *Sstr4* mRNA expression in the pituitary gland, lungs, and thymus, respectively. The dynamics of *Sstr4* mRNA expression were investigated using the real-time PCR method and ΔΔCt method. Data are shown as mean ± standard error, with n = 7–8 for each group. Individual data points are also shown in the graph. Investigations of significant differences between the single-stress group, chronic-stress group, and their respective control groups were conducted using the Mann–Whitney U test. *P < 0.05. ***P < 0.01. Significant differences between the single-stress and chronic-stress groups were not investigated.

We performed real-time polymerase chain reaction (PCR) to investigate the dynamics of *Sstr4* mRNA expression in the pituitary gland, lungs, and thymus (Fig. 2). In the pituitary gland, a significant decrease in *Sstr4* mRNA expression was observed in the single-stress group compared to that in its corresponding control group (P = 0.0022). A significant decrease in *Sstr4* mRNA expression was observed in the chronic-stress group compared to that in its corresponding control group (P = 0.0181, Fig. 2a).

In the lungs, a significant decrease in *Sstr4* mRNA expression was observed in the single-stress group compared to that in its corresponding control group (P = 0.0124). No significant difference in *Sstr4* mRNA expression was observed between the chronic-stress and control groups (Fig. 2b).

No significant difference in *Sstr4* mRNA expression was observed in the thymus between the single-stress and corresponding control groups. A significant increase in *Sstr4* mRNA expression was observed in the chronic-stress group compared to that in its corresponding control group (P = 0.0313, Fig. 2c).

### SSTR4 protein expression

**Figure 3:**
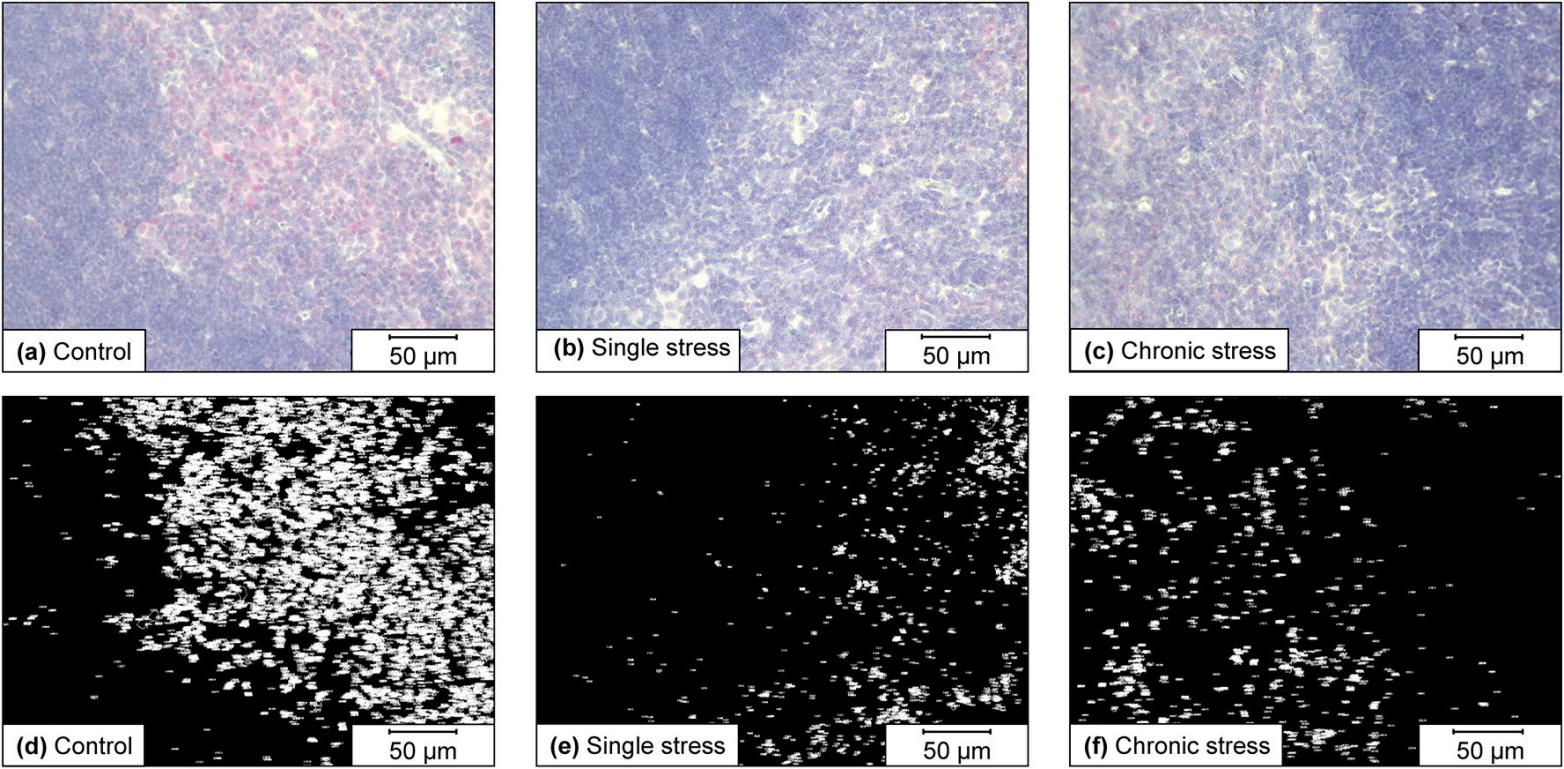
Representative images of the thymus from the control, single-stress and chronic-stress groups after immunostaining with anti-SSTR4 antibodies and particle analysis using Fiji. (**a**–**c**) Histology of the mouse thymus in the control group (**a**), single-stress (**b**), and chronic-stress ((**c**) groups after immunostaining with an anti-SSTR4 antibody using BZ-9000. The red-colored areas indicate positivity for the anti-SSTR4 antibody. (**d**–**f**) The resulting image obtained after performing the particle analysis of the image in ((**a**–**c**), respectively) using Fiji. The magnification of the post-analysis image is identical to that of the pre-analysis image.

We investigated the dynamics of SSTR4 expression in the thymus by immunostaining with an anti-SSTR4 antibody and analyzing the percentage area of the anti-SSTR4 antibody-positive region (Fig. 3).

**Figure 4:**
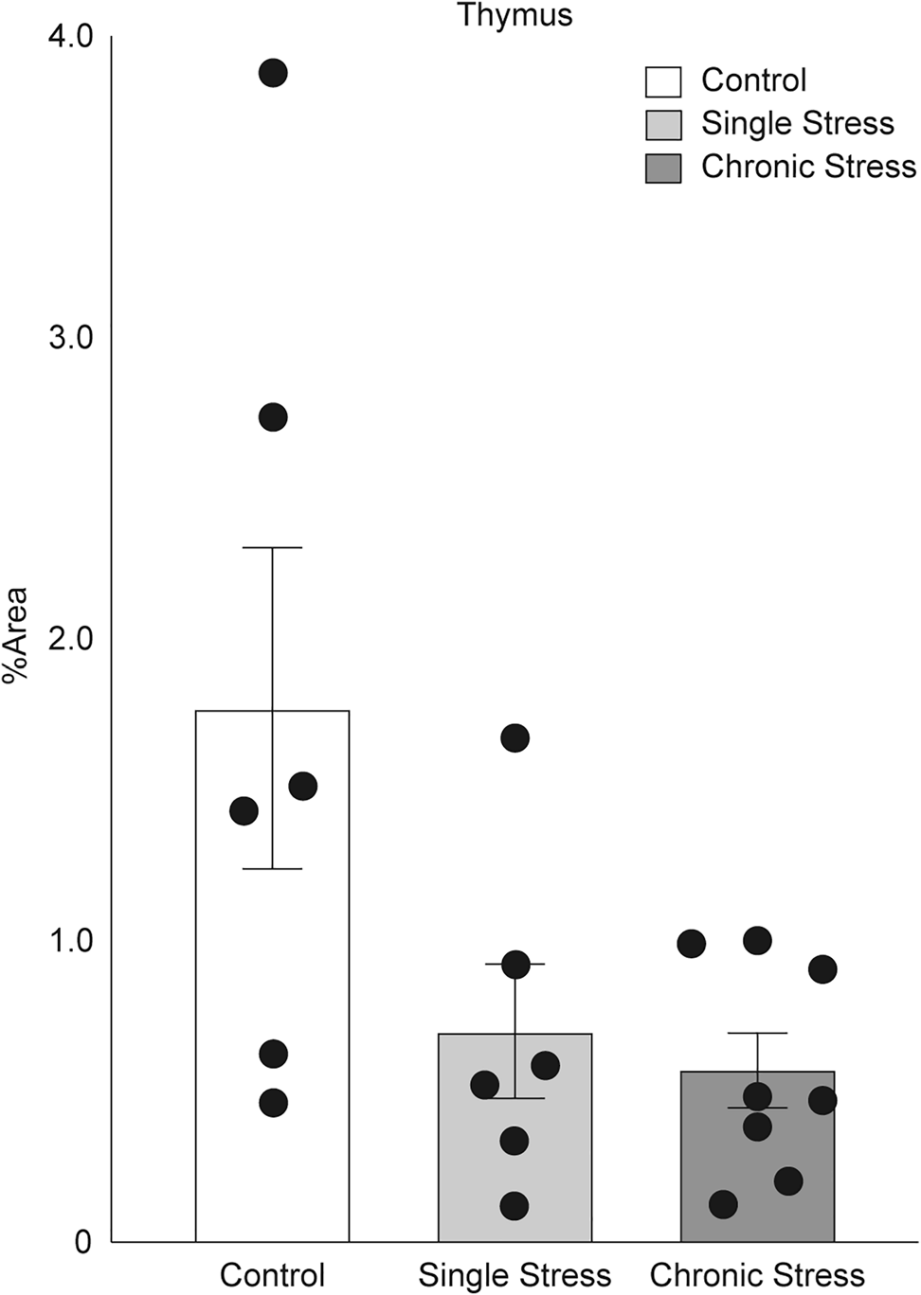
Dynamics of SSTR4 protein expression in the thymus in the control, single-stress, and chronic-stress groups. The graph shows the area percentage (% Area) of the anti-SSTR4 antibody-positive area obtained by conducting particle analysis with Fiji. Data are shown as mean ± standard error, with n = 6–8 for each group. Individual data points are also shown in the graph. Investigations of significant differences between the control, single-stress, and chronic-stress groups were conducted using the Kruskal–Wallis test (P = 0.0963). The mean values were 1.77135% in the control group, 0.69452145% in the single-stress group, and 0.5715375% in the chronic-stress group.

In the thymus, no significant changes in SSTR4 protein expression were observed in the chronic- and single-stress groups compared to that in the control group. However, the mean % area was reduced by < 50% in the chronic- (0.5715375%) and single-stress (0.69452145%) groups compared to the control group (1.77135%); however, the differences were not significant (P = 0.0963; Fig. 4).

## Discussion

SSTR4, a G-protein-coupled receptor, is a member of the SSTR family, comprising five proteins (SSTR1–5). These proteins interact with somatostatin and are involved in several biological effects. Somatostatin plays a key role in inhibiting the secretion of hormones like growth hormones, prolactin, insulin, and secretin^14–16^. In addition, somatostatin is associated with depression and responsiveness to drug treatment^18–20^. For instance, somatostatin levels in the cerebrospinal fluid (CSF) of patients with depression are reduced^18^. Administering somatostatin into the cerebral ventricles of rats has anxiolytic and antidepressant-like effects^19^. Moreover, patients with low somatostatin levels in the CSF respond positively to treatment with the antidepressant nimodipine^18^. The therapeutic potential of SSTR4 for anxiety and depressive disorders has also been reported^21^. Several indirect and direct studies have demonstrated the association between SSTR4 and depression, which also relates to chronic stress^4–7^. In this study, we tested the hypothesis that SSTR4 expression is regulated by multiple mechanisms during chronic stress loading to unveil its potential as a diagnostic marker for chronic stress.

In this study, we confirmed the effectiveness of administering restraint stress treatment once daily for 1 h by measuring the corticosterone concentration in mouse plasma 1 h after treatment. The results showed a significant increase in plasma corticosterone concentration in the single-stress group compared to that in the corresponding control group. Moreover, no significant changes in plasma corticosterone concentrations were observed in the chronic-stress group compared to the corresponding control group.

Corticosterone secretion increases in rodents, such as mice, after stress^23,26^. Moreover, fluctuation in corticosterone levels under chronic stress is well established.

Specifically, in rodents, adaptation of the hypothalamus–pituitary–adrenal axis to repeated or chronic homotypic stressors is well-documented^26–30^. A previous study has shown that under repeated homotypic stress, post-stress plasma corticosterone levels are lower on the eighth day than those on the first day of stress induction^28^. Chronic homotypic stress with chronic subordinate colony housing (CSC) results in a significant elevation of plasma corticosterone levels only on the second day of stress initiation compared to pre-stress levels, but no significant difference was observed thereafter^29^. Applying homotypic restraint stress, as done in our study with mice, may induce adaptation, potentially leading to a decrease in plasma corticosterone concentration after one week compared to those on the first and second days of stress initiation. Furthermore, plasma corticosterone levels have been shown to increase during restraint stress treatment but do not persist post-treatment^26^, and chronic homotypic stress may not alter basal corticosterone secretion^26,30^. Consistent with these findings, our study revealed no significant changes in plasma corticosterone concentrations between the chronic stress and control groups 1 h after stress treatment. These findings suggest that the high corticosterone state does not persist in vivo, even if chronic restraint stress is applied. Therefore, the lack of significant differences in the plasma corticosterone concentration 1 h after the end of stress in the chronic-stress group does not signify that the restraint stress treatment used in the present study was inappropriate. Notably, a significant increase in plasma corticosterone concentration was observed in the single, 1 h restraint stress group compared to that in the control group. Given that rodents, such as mice, exhibit increased corticosterone secretion after stress, the restraint treatment performed in this study was conceivably an appropriate stress load for a single-stress case. Furthermore, following chronic restraint stress treatment, a significant decrease in the relative thymus weight was observed between the chronic-stress and control groups, and weight loss was observed only in mice of the chronic-stress group.

Reber et al*.*^29^ reported that, following the application of chronic psychological stress using CSC, mice exhibit weight loss, relative thymus weight loss, and relative adrenal weight increase. They reported that reduced body weight gain is a characteristic of chronic stress. In contrast, thymus atrophy (relative thymus weight loss) and adrenal hypertrophy (relative adrenal weight gain) are additional stress indicators. Zelena et al*.*^31^ reported that weight loss, relative thymus weight loss, and relative adrenal weight increase were observed after applying stress through multiple restraints. Therefore, weight loss and relative thymus weight loss, which are signs of chronic stress, were observed in the chronic-stress group in the present study, suggesting that the restraint stress used in this experiment also functioned as chronic stress. In summary, the restraint stress treatment used in the present study was considered appropriate, even during chronic stress.

In the present study, we investigated the dynamics of SSTR4 expression during single- and chronic-stress loading. Furthermore, there are no confirmed reports of SSTR4 expression in the thymus of mice with MGI, and no studies have reported SSTR4 expression in the thymus. We confirmed the expression of *Sstr4* mRNA using real-time PCR, and an anti-SSTR4 antibody-positive region was observed using immunostaining. Therefore, to our knowledge, this is the first study to report SSTR4 expression in the thymus.

*Sstr4* expression at the mRNA level increased in the thymus only in the chronic-stress group; this parameter could lead to the diagnosis of chronic stress. Furthermore, a significant decrease in *Sstr4* mRNA expression level was observed in the pituitary gland in both the single and chronic-stress groups compared to their control groups. Therefore, investigating *Sstr4* mRNA expression levels in the pituitary gland may indicate the presence or absence of stress, regardless of chronic or single stress. A significant decrease in *Sstr4* mRNA expression was observed in the lungs of the single-stress group compared to that in the control group. Still, no significant differences were observed between the chronic-stress group and its corresponding control group. Thus, investigating *Sstr4* mRNA expression levels in the lungs may provide evidence that the stress-loading period is short.

The dynamics of *Sstr4* mRNA expression may differ depending on the organ, and it is possible to estimate the stress-loading period by investigating *Sstr4* mRNA expression in each organ. Specifically, in cases where the *Sstr4* mRNA expression level is increased in the thymus but unchanged in the lungs, the individual is most likely experiencing prolonged stress. Moreover, in cases where the *Sstr4* mRNA expression level is unchanged in the thymus but decreased in the lungs, the individual has most likely been under stress for only a short period. Furthermore, factors other than stress may also be considered in cases where the *Sstr4* mRNA expression level is increased in the thymus but remains unchanged in the pituitary gland.

In the present study, the mice used for investigating *Sstr4* mRNA expression were procured at eight weeks of age, subjected to restraint stress for 1 week starting at nine weeks of age, and euthanized at ten weeks, after which each organ was collected. Mice are weaned 3–4 weeks after birth, reach puberty at approximately 42 days, and become sexually mature at 8–12 weeks of age (average 10 weeks), developing into adults^32^. Thus, the mice used in this experiment were early or mature adults. Moreover, *Sstr4* mRNA expression is expected to fluctuate in each organ in a manner similar to the results of the present experiment, even in cases of IPV or elder abuse. Thus, SSTR4 could be a potentially useful marker for diagnosing the stress-loading period. However, further consideration is needed regarding elder abuse because the mice in this experiment were 10 weeks old at the time of euthanasia. In contrast, the development of aging in mice begins at 10–15 months, with ≥ 18 months defined as aging^32^. Therefore, although the present results may apply to elder abuse cases, additional experiments in aged mice are necessary to confirm these findings.

In the present study, the mean SSTR4 expression in the thymus (% area) was reduced by less than 50% in both the chronic and single-stress groups compared to that in the control group. Stress appeared to decrease SSTR4 protein expression. However, the differences between the chronic-stress and control groups and between the single-stress and control groups were not significant. This observation could be caused by the high variability in the control group data. In contrast, the single and chronic-stress groups showed relatively little variation in the mean % area. The data were clustered at lower values relative to the mean of the control group (control group: range, 0.4657–3.8731%; standard deviation (SD), 1.3066; single-stress group: range, 0.124–1.6727%; SD, 0.5481; chronic-stress group: range 0.1259–1.0001%, SD, 0.3500). Therefore, it may be useful to set a cutoff value when examining the protein expression levels of SSTR4 in the thymus. For example, according to the results of this experiment, if the % area was above 1.0001%, the mice were most likely not subjected to chronic-stress load. If the % area was above 1.6727%, the mice were not under a stress load, as they may not have been subjected to single stress either. However, the SSTR4 expression level in the thymus may be low, even in the control group. Therefore, the protein expression level of SSTR4 in the thymus does not indicate that the mice were stressed. In addition, there were no significant differences between the single and chronic-stress groups, and SSTR4 protein expression levels were relatively similar. Therefore, it was difficult to determine the stress period by examining the SSTR4 protein expression levels in the thymus. The lack of significant differences in SSTR4 protein expression in this experiment may be caused by the high variability of the data in the control group, which may be attributed to the small number of mice involved in the experiment. We believe that significant differences between the control groups and each stress group may become apparent as the number of mice increases. In addition, if the *Sstr4* mRNA and protein expressions are related, SSTR4 expression may fluctuate in the lungs and pituitary glands, which we intend to investigate in the future.

In addition, the changes in SSTR4 expression dynamics observed in this study may reflect chronic stress-induced mental illnesses (e.g., anxiety and mood disorders) and depression-like behaviors, as the increase in plasma corticosterone levels does not persist in vivo after chronic-stress loading. However, further investigation of this hypothesis is needed, as we did not examine whether the mice exhibited mental illness disorders or depression-like behaviors.

This study had several limitations. First, SSTR4 expression under stressors other than restraint stress, such as forced swimming, heat, isolation, and social defeat stress, was not investigated. Although this study indicates that restraint stress may be associated with SSTR4 expression, it is impossible to determine whether SSTR4 expression varies similarly under other stresses. However, it is also important to determine the response of SSTR4 to various stressors. For example, by examining whether physical stress (e.g., forced swimming and heat stress) or non-physical stress (e.g., isolation and social defeat stress) alters SSTR4 expression, it may be possible to determine whether mental or physical stress has a greater influence on the changes in SSTR4 expression. Second, this experiment was conducted only in mice, and it is unknown whether SSTR4 can act as a stress marker in humans.

In conclusion, the study findings suggest that SSTR4 expression may change in the thymus, lungs, and pituitary gland in relation to stress and the stress-loading period and that the dynamics of SSTR4 expression differ in each organ. Among studies on SSTR4 and stress- or depression-like behavior, to our knowledge, the dynamics of SSTR4 expression outside the brain have yet to be investigated^17,21,24,25^., and the results of the present study constitute novel findings. Thus, SSTR4 could serve as a marker for stress and estimation of the stress-loading period.

## Methods

### Animals

In this study, we used pathogen-free six-week-old and eight-week-old BALB/c male mice procured from SLC, Inc. (Shizuoka, Japan). The mice were housed in plastic cages. The rooms were exposed to a 12 h light/dark cycle (lights on at 7:00 AM, lights off at 7:00 PM) and maintained at 23 ± 2 °C. The experiment was initiated after > 1 week of acclimatization of the animals to the experimental environment. All experiments in this study had 2–3 mice of the same group in each cage. In addition, for each experiment, all mice were maintained in the same room, even during restraint treatment, to avoid differences owing to the experimental environment. In this study, the mice were grouped on the day of delivery or the next day to maintain similar average body weights in each group for all experiments; randomization and blinding were not performed. The study protocol was approved by the Animal Care Committee of Nagasaki University (approval no. 0811100715 and 2006251645). Mice were treated in accordance with the Fundamental Guidelines for Proper Conduct of Animal Experiment and Related Activities in Academic Research Institutions, under the jurisdiction of the Ministry of Education, Culture, Sports, Science and Technology of Japan. The authors complied with the ARRIVE guidelines.

### Evaluating the efficacy of restraint stress treatment

Before experiment initiation, we measured plasma corticosterone concentrations to investigate whether restraint stress was as effective as stress. Six-week-old BALB/c male mice were subjected to restraint stress treatment by placing them in a 50 mL falcon tube perforated for ventilation once daily for 1 h. The mice were restrained and could not consume food or drink water. Restraint stress treatment began between 7:00 AM and 9:00 AM, except on blood sampling days. Restraint treatment was initiated between 8:00 and 9:00 AM on the day of blood collection. It should be noted that this is a preliminary experiment conducted to validate the restraint experiments, and the choice to use six-week-old mice was based on our familiarity with this age group. Furthermore, the sample size could not be calculated due to the unpredictable change in plasma corticosterone concentrations during this restraint stress. Nevertheless, it is well-known that plasma corticosterone levels fluctuate in response to stress. Hence, we used a sample size of 5–6 mice per group for this experiment, which we deemed sufficient for statistical analysis.

In this experiment, 21 six-week-old male mice were used. We established a chronic-stress group (n = 6) wherein mice were repeatedly treated with restraint stress for one week; a single-stress group (n = 5) wherein mice were treated with restraint stress only once; and control groups (n = 5 each) wherein mice were not treated with restraint stress. The effectiveness of restraint stress was investigated using four groups of mice: chronic-stress, single-stress, and their respective control groups.

The stress and control groups were euthanized through expert decapitation on the same day. Decapitation was performed using decapitation scissors, and the scissors were washed after each use. The breeding room and the sampling room were separate, and only one mouse was present in the sampling room during decapitation as decapitation could potentially induce stress in another mouse present in the room.

The mice in each stress group were euthanized 1 h after restraint treatment, and mice in the control groups were euthanized immediately before the mice in the stress groups, and their blood was collected. The single-stress group was subjected to stress on the day of euthanasia. Plasma was collected after the centrifugation of each blood sample. The corticosterone concentration in each plasma sample was measured using a Cayman EIA kit 500,655 (Cayman Chemical Company, Ann Arbor, MI, USA), and significant differences in corticosterone concentrations were examined. Mice who died during restraint procedures before euthanasia and those whose blood could not be collected were set to be excluded. However, none of the mice were excluded.

### Restraint stress model

A restraint stress model was adopted for the study using eight-week-old male BALB/c mice, which were used for all subsequent experiments. Sample size calculation was not feasible due to the unknown kinetics of *Sstr4* expression. Therefore, the sample size we intended to use was 6–8 per group, which corresponds to the number of mice used for Fos immunostaining in a previous study^17^. However, certain organs, like the pituitary and thymus, are small or challenging to harvest due to chronic stress; hence, the maximum sample size was set at 9. Consequently, the sample size employed was 6–9 per group to accommodate the potential failure to harvest.

The mice were placed in a 50-mL falcon tube and restrained once a day for 8 h. Stress treatment was initiated between 7:00 AM and 9:00 AM on non-organ collection days. On the day of organ collection, restraint treatment was initiated between 8:00 and 10:00 AM. Mice were divided into chronic-stress (repeated restraint stress for one week), single-stress (restraint stress only once), and control groups (no restraint stress). Experiments were initiated after acclimating mice to the experimental environment for approximately one week. All groups were euthanized at 10 weeks of age. We selected this age group because we aimed to apply the findings of this experiment to IPV, elder abuse, and similar applications, where sexually mature mice were preferred. Mice are known to reach sexual maturity at an average age of 10 weeks^32^. Therefore, only eight-week-old mice were used in all subsequent experiments.

### Organ sampling and thymus weight measurements

Mice treated with restraint stress were euthanized via decapitation by an expert 1 h after the final restraint stress treatment ended. The mice in the control group were euthanized at a time similar to that for the stress group. This euthanasia method was consistent with the one used in the experiment to evaluate the efficacy of the restraint stress treatment. Organs were collected after euthanasia, which included the pituitary gland, thymus, and lungs. For the analysis of *Sstr4* mRNA expression dynamics, 30 mice were divided into the following four groups: chronic-stress (n = 8), single-stress (n = 7), control for chronic stress (n = 8), and control for single stress (n = 7). To analyze SSTR4 protein expression dynamics, we prepared and investigated the following three groups of mice (21 mice in total): the chronic-stress group (n = 9), the single-stress group (n = 6), and the control group (n = 6). The thymus was collected from the mice.

In addition, mouse models were created to measure changes in body and thymus weights induced by restraint stress. These mice were subjected to the same grouping, breeding room, breeding environment, restraint treatment, and euthanasia as those used for the *Sstr4* mRNA expression dynamics analysis. A total of 25 mice were grouped into chronic-stress (n = 6), single-stress (n = 7), and two respective control (n = 6 each) groups. Body weights were measured one week before euthanasia (before stress) and one day before euthanasia (after stress). Mice in the chronic-stress group were weighed before the start of restraint stress on the same day, whereas mice in the single-stress group and the respective controls were weighed immediately before or after those in the chronic-stress group were weighed. Thymus weight was measured after euthanasia. Based on these data, we examined whether there were any significant differences in changes in body weight and relative thymic weight.

Mice who died during the restraint procedure or before euthanasia and those from which the target organs could not be collected were excluded from the experiments. In the SSTR4 protein expression analysis, one mouse was excluded from the chronic-stress group because thymus collection could not be performed. No mice were excluded from the other experiments. Therefore, for the SSTR4 protein expression kinetics analysis, eight, six, and six mice were included in the chronic-stress, single-stress, and control groups, respectively (20 mice in total).

In this experiment, although we made efforts to minimize the difference in euthanasia time between the stress and control groups, the variations were still present, ranging from 2 to 3 h. As the diurnal variation in SSTR4 is currently unknown, we evaluated a control group along with each restraint stress group to account for any potential diurnal variation in *Sstr4*, except for the experiment analyzing SSTR4 expression. *Sstr4* mRNA levels revealed no marked differences between the control groups; we speculated no considerable differences in SSTR4 expression, at least during the experiment. Therefore, the experiment for SSTR4 expression analysis was conducted in three groups: chronic-stress, single-stress, and control.

### Analysis of mRNA expression

Real-time PCR was performed to investigate *Sstr4* mRNA expression. Each organ was soaked in RNAlater Solution (Thermo Fisher Scientific, Waltham, MA, USA) after collection, stored overnight at 4 °C (approximately 12–14 h), and then stored at − 80 °C until use (up to 29 weeks). The RNeasy Mini Kit (Qiagen, Hilden, Germany) was used for total RNA extraction according to the manufacturer’s protocol (Qiagen). The extracted total RNA was stored at − 30 °C until use and reverse-transcribed using the PrimeScript RT Reagent Kit (Takara Bio, Kusatsu, Japan). Real-time PCR was conducted with a 10 µL reaction system using SYBR Premix EX Taq II (Takara Bio) and a Thermal Cycler Dice Real-Time System (Takara Bio). The amplification mix composition and thermal conditions were determined according to the manufacturer’s protocol. Primers to amplify *Sstr4* mRNA and ribosomal protein S18 (*Rps18*), used as an internal control, were purchased from Takara Bio. The primer sequence for *Sstr4* was (F: 5′-TCCTACGCTATGCCAAGATGAAGA-3′, R: 5′-ATGGCACGCTGAGCATGAAG-3′). The primer sequence for *Rps18* was (F: 5′-TTCTGGCCAACGGTCTAGACAAC-3′, R: 5′-CCAGTGGTCTTGGTGTGCTGA-3′). We conducted a comparative quantification of *Sstr4* mRNA expression using the ∆∆CT method.

### Protein expression analysis

We used immunostaining to analyze the expression dynamics of SSTR4. The thymus of each group was placed in 4% paraformaldehyde/phosphate buffer (Wako, Osaka, Japan) immediately after collection and fixed overnight at 4 °C (approximately 12–14 h). Subsequently, the thymus sections were embedded in paraffin and stored (up to 8 weeks) in the dark at room temperature (15–26 °C).

A microtome was used to slice the paraffin-embedded specimen into 4 µm-thick sections. Immunostaining was conducted using an anti-SSTR4 rabbit polyclonal primary antibody (NB100-74,539, Funakoshi Co., Ltd., Tokyo, Japan), and immunostaining was performed using a standard protocol (Abcam, Cambridge, UK). An antigen-activating solution was prepared using an aqueous citric acid solution, and each section was subjected to antigen activation using heat. The sections were then blocked with blocking solution (Tris-buffered saline containing 1% casein, 10% normal goat serum, and 0.1% Triton X-100) at room temperature (20–25 °C) for 1 h. Next, a primary antibody solution (blocking solution: anti-SSTR4 rabbit polyclonal antibody = 200:1) was placed on the sections and incubated overnight at 4 °C (approximately 14–18 h). The following day, the sections were washed and incubated with a secondary antibody solution, which was prepared using goat anti-rabbit immunoglobulin (Ig)G H&L alkaline phosphatase (Abcam) (blocking solution: goat anti-rabbit IgG H&L alkaline phosphatase, 500:1). Incubation was performed in a microwave rapid sample-processing device MI-77 (Azumaya Medical Instruments, Tokyo, Japan), and intermittent microwave irradiation was applied for 20 min. A Histofine First Red II Substrate Kit (Nichirei Bioscience Inc., Tokyo, Japan) was used as the color-developing solution. Counterstaining was performed using hematoxylin.

### Observation and assessment

Immunostaining analysis was conducted using BZ-9000 (Keyence, Osaka, Japan) and Fiji^33^, one of the image analysis software distributors, ImageJ. Each section was initially observed under a microscope, and 10 images were taken for each section with a BZ-9000 microscope using a 40 × objective lens. Regions that were unsuitable for analysis, such as those with no tissue, were deleted from the image. No other processing was performed before analyzing the images using Fiji. Next, Fiji was used to divide the images into independent red, green, and blue images. After division processing, the green image was subtracted from the red image, and binarization was conducted with the lower and upper limits of the thresholds set to 20 and 255, respectively. We conducted particle analysis on the binarized images to calculate the percentage area (% area) of the particles in the analysis range (i.e., the percentage area of the anti-SSTR4 antibody-positive region).

### Statistical analyses

The experimental plasma corticosterone data for evaluating the efficacy of the restraint stress treatment were examined for significant differences using Welch's *t*-tests. Data on changes in body weight of mice before and after restraint stress treatment, thymus weights, and relative thymus weights were obtained by organ sampling and thymus weight measurements. Significant differences in these data were analyzed using Welch's t-test. For the expression dynamics of *Sstr4* mRNA, the ∆∆Ct method was used to calculate relative SSTR4 expression. We further examined whether there were significant differences in relative *SSTR4* expression using the Mann–Whitney U test. The percentage area of the immunostained anti-SSTR4 antibody-positive region (% area) was analyzed, and the expression kinetics of the SSTR4 protein were examined using the Kruskal–Wallis test. All statistical analyses were performed using the JMP Pro 16^®^ software (SAS Institute, Cary, NC, USA). A P-value of < 0.05 was considered statistically significant.

## Data Availability

The data supporting the findings of this study are openly available on Figshare at http://doi.org/10.6084/m9.figshare.23560461.
